# TIMM23 overexpression drives NSCLC cell growth and survival by enhancing mitochondrial function

**DOI:** 10.1038/s41419-025-07505-3

**Published:** 2025-03-13

**Authors:** Jianhua Zha, Jiaxin Li, Hui Yin, Mingjing Shen, Yingchen Xia

**Affiliations:** 1 Department of Thoracic Surgery, The First Affiliated Hospital of Nanchang University, Nanchang, China; 2 Jiangxi Hospital of China-Japan Friendship Hospital, National Regional Center for Respiratory Medicine Nanchang, Nanchang, China; 3 Department of Pharmacy, Jiangxi University of Chinese Medicine, Nanchang, China; 4 Department of Thoracic Surgery, The First Affiliated Hospital of Shaoyang University, Shaoyang, China; 5 Department of Thoracic and Cardiac Surgery, The Second Affiliated Hospital of Soochow University, Suzhou, China; 6 Department of Thoracic Surgery, The First Affiliated Hospital of University of Science and Technology of China (USTC), Division of Life Sciences and Medicine, University of Science and Technology of China, Hefei, China

**Keywords:** Non-small-cell lung cancer, Targeted therapies

## Abstract

Mitochondrial hyperfunction is implicated in promoting non-small cell lung cancer (NSCLC) cell growth. TIMM23 (translocase of inner mitochondrial membrane 23) is a core component of the mitochondrial import machinery, facilitating the translocation of proteins across the inner mitochondrial membrane into the matrix. Its expression and potential functions in NSCLC were tested. Comprehensive bioinformatic analysis revealed a strong correlation between *TIMM23* overexpression and adverse clinical outcomes in NSCLC patients. Single-cell RNA sequencing data further corroborated these findings, demonstrating elevated *TIMM23* expression within the cancer cells of NSCLC mass. Subsequent experimental validation confirmed significantly increased *TIMM23* mRNA and protein levels in locally-treated NSCLC tissues compared to matched normal lung tissues. Moreover, TIMM23 expression was consistently elevated across multiple primary/established NSCLC cells. Silencing or ablation of TIMM23 via shRNA or CRISPR/Cas9 in NSCLC cells resulted in impaired mitochondrial function, characterized by reduced complex I activity, ATP depletion, mitochondrial membrane potential dissipation, oxidative stress, and lipid peroxidation. These mitochondrial perturbations coincided with attenuated cell viability, proliferation, and migratory capacity, and concomitant induction of apoptosis. Conversely, ectopic overexpression of TIMM23 significantly enhanced mitochondrial complex I activity and ATP production, promoting NSCLC cell proliferation and motility. In vivo, intratumoral delivery of a TIMM23 shRNA-expressing adeno-associated virus significantly suppressed the growth of subcutaneous NSCLC xenografts in nude mice. Subsequent analysis of tumor tissues revealed depleted TIMM23 expression, ATP reduction, oxidative damage, proliferative arrest, and apoptotic induction. Collectively, these findings establish TIMM23 as a critical pro-tumorigenic factor in NSCLC, highlighting its potential as a prognostic biomarker and therapeutic target.

## Introduction

Lung cancer constitutes a significant proportion of the global cancer burden, comprising over 13% of newly diagnosed malignancies [1, 2]. The United States alone witnesses an annual incidence close to 200,000 cases [3]. Non-small cell lung cancer (NSCLC) represents the predominant histological subtype and remains a primary cause of mortality worldwide. Characterized by aggressive metastatic behavior, advanced NSCLC presents a substantial clinical challenge [4, 5]. The 5-year survival rates for NSCLC are stage-dependent: approximately 60% for localized disease, around 35% for regional disease, and approximately 6-7% for distant metastatic disease [3–5].

Recent years have witnessed substantial advancements in elucidating the molecular underpinnings of NSCLC pathogenesis [6–10]. For instance, the identification of somatic alterations in genes such as *epidermal growth factor receptor* (*EGFR*), *B-Raf proto-oncogene, serine/threonine kinase* (*BRAF*), and mesenchymal epithelial transition (*MET*), as well as *anaplastic lymphoma kinase* (*ALK*), *rearranged during transfection* (*RET*) has transformed NSCLC diagnostic and therapeutic paradigms [4, 11]. Targeted therapies directed against these molecular aberrations, using specific inhibitors or monoclonal antibodies, have demonstrated clinical efficacy [6-10]. Moreover, current immunotherapy for NSCLC primarily employs immune checkpoint inhibitors targeting PD-1/PD-L1 and CTLA-4 (cytotoxic t lymphocyte-associated protein 4) pathways, demonstrating significant clinical benefits in specific patient populations [12–14]. However, despite these therapeutic advances, outcomes for patients with recurrent, metastatic, or refractory NSCLC remain dismal [4, 11, 15]. Consequently, the identification of novel therapeutic targets and the development of innovative treatment modalities for this patient population constitute an urgent unmet clinical need. [8, 16–18].

Mitochondria are indispensable organelles orchestrating a myriad of cellular processes, including oxidative phosphorylation (OXPHOS), ATP generation, amino acid metabolism, macromolecular biosynthesis, fatty acid oxidation, and ionic homeostasis [19–23]. Moreover, they serve as pivotal platforms for signal transduction and apoptotic regulation [19–23]. The metabolic reprogramming characteristic of cancer cells is often accompanied by profound alterations in mitochondrial function [19, 24]. To sustain their rapid proliferation, cancer cells exhibit enhanced bioenergetic capacity, primarily driven by augmented ATP production through mitochondrial respiration [19–23]. In the context of NSCLC, mitochondrial hyperfunction has been implicated as a critical hallmark of tumorigenesis and progression [6, 25–27]. For example, elevated heme biosynthesis or uptake can potentiate mitochondrial OXPHOS, thereby fueling tumor growth. Conversely, pharmacological inhibition of heme metabolism suppressed mitochondrial respiration, consequently impeding NSCLC cell growth [7, 23]. Delineating the precise molecular mechanisms underlying mitochondrial alterations in NSCLC remains a focal area of ongoing investigation [6, 25].

TIMM23 (translocase of inner mitochondrial membrane 23) is a pivotal component of the mitochondrial import machinery, integral to the TIM23 complex [28–30]. This complex mediates the translocation of preproteins across the inner mitochondrial membrane into the mitochondrial matrix or facilitates their insertion into the inner membrane [28–30]. Such processes are essential for mitochondrial biogenesis and function, ensuring the proper import of cytoplasmically synthesized proteins into the mitochondria [28–30]. These imported proteins are crucial for mitochondrial activities related to energy production, metabolic processes, and the regulation of apoptosis [28–30]. Recent studies have also discovered that TIMM23 is a crucial component of a PINK1-containing protein complex involved in mitochondrial quality control [31–33]. The expression and functional role of TIMM23 in NSCLC remain unexplored and is the primary focus of this study.

## Materials and methods

### Reagents

All cell culture reagents, including fetal bovine serum (FBS) and medium, were procured from Hyclone (Logan, UT). Fluorescence dyes, including JC-1, DAPI, EdU, TUNEL, CellROX, and DCF-DA, were purchased from Thermo Fisher Invitrogen Scientific (Carlsbad, CA, USA). A Histone-bound DNA ELISA Kit was obtained from Roche Diagnostics (Indianapolis, IN, USA). The antioxidant N-acetylcysteine (NAC), cell permeable ATP (ATPγS) and all other chemicals utilized in this study were provided by Sigma (St. Louis, MO, USA) “Transwell” chambers were acquired from Corning Incorporated (New York, NY).

### Cells

The human lung adenocarcinoma cell line, A549, was obtained from the Shanghai Institute for Biological Sciences (Shanghai, China) and maintained in RPMI-1640 medium supplemented with 10% FBS. Primary human NSCLC cells (“pNSCLC-1”, “pNSCLC-2”, and “pNSCLC-3”, “p” stands for primary) and primary lung epithelial cells were isolated from written-informed consent patients and donors as previously reported [26, 34, 35]. These primary cells were cultured under conditions described in the early studies [34, 35]. All experimental protocols involving human cells were approved by the Ethics Committee of Nanchang University and adhered to the guidelines of the Declaration of Helsinki. To ensure cell line authenticity and integrity, routine testing for mycoplasma contamination, microbial contamination, and genetic stability (short tandem repeat profiling) was conducted.

### Human tissues

Twenty pairs of human NSCLC tumor (“T”) and corresponding adjacent normal lung epithelial (“N”) tissues were obtained from patients diagnosed with advanced-stage (III-IV) LUAD disease at our institution. Upon surgical resection, specimens were immediately frozen in liquid nitrogen to preserve tissue integrity and subsequently homogenized in a lysis buffer containing protease inhibitors [34, 35]. Alternatively, the tissue slides were subjected to standard immunohistochemistry (IHC) staining procedures to examine TIMM23 staining, with IHC scores recorded by two independent experts. All patient-derived materials and associated clinical data were handled in strict accordance with ethical guidelines. Lung cancer samples were collected from written-informed consent patient with approval from the Ethics Committee of the First Affiliated Hospital of Nanchang University (NO. (2024) CDYFYYLK (08-011)). Research conduct adhered to the principles outlined in the Declaration of Helsinki.

### TIMM23 silencing

NSCLC cells were cultured in complete (FBS containing) medium supplemented with polybrene to 60% confluence prior to lentiviral transduction with TIMM23 shRNA constructs (Genechem, Shanghai, China) at a multiplicity of infection (MOI) of 11-12. Following a 48 h incubation, puromycin selection was implemented to establish stable shRNA-expressing cells within six passages. Three distinct shRNA sequences targeting TIMM23 were employed: shTIMM23-1, shTIMM23-2, and shTIMM23-3. Control cells were transduced with a scrambled shRNA (shC) lentivirus [26]. Continuous monitoring of TIMM23 mRNA and protein levels was conducted in all cells. For in vivo studies, the shTIMM23-3 sequence or the shC sequence was incorporated into a previously described adeno-associated virus (AAV) vector to generate recombinant AAV particles [26].

Generation of TIMM23 knockout (KO) NSCLC cells. To generate TIMM23 knockout (KO) NSCLC cells, CRISPR/Cas9-mediated gene editing was employed [26]. NSCLC cells were cultured in complete medium supplemented with polybrene to 55% confluence prior to lentiviral transduction with a Cas9-expressing vector, as previously described [26]. Stable Cas9-expressing cells were subsequently infected with a lentiviral vector harboring a CRISPR/Cas9-TIMM23-KO puromycin resistance cassette. This construct contained either of two single guide RNAs (sgRNAs) targeting distinct TIMM23 loci: koTIMM23-sg1 or koTIMM23-sg2, obtained from Genechem (Shanghai, China). Puromycin selection yielded stable TIMM23 KO cell clones, which were subsequently isolated in 96-well plates. Efficient TIMM23 KO was validated by targeted deep sequencing of the sgRNA target site and Western blotting analysis to verify protein absence. Control cells were generated through identical procedures using a lentiviral vector encoding a CRISPR/Cas9-empty control cassette (“Cas9-C”) as previously reported [26].

### TIMM23 overexpression

To achieve robust and sustained overexpression of TIMM23, NSCLC cells were subjected to lentiviral transduction employing a construct (GV369, Genechem, described early [26]) encoding the full-length *TIMM23* cDNA sequence at a multiplicity of infection (MOI) of 10. Post-infection, cells were cultured for 60 h to permit efficient viral integration and TIMM23 expression. Subsequently, stable cells exhibiting constitutive TIMM23 overexpression were established through rigorous puromycin selection spanning six consecutive passages. To ensure the fidelity and consistency of TIMM23 overexpression, mRNA and protein expression levels were monitored periodically within these stable cells.

Measuring mitochondrial complex I activity and ATP contents. Mitochondrial complex I activity was determined spectrophotometrically in cell and tissue lysates using a commercial colorimetric assay kit (Sigma). The enzymatic conversion of NADH to NAD+ was monitored at 380 nm. A decrease in absorbance at this wavelength corresponded to complex I activity. Intracellular ATP levels were assessed in described cellular/xenograft lysates using a colorimetric assay kit (Sigma) according to the manufacturer’s protocol. For each analysis, 20 μL of lysates containing a standardized protein concentration of 20 μg were employed.

Lipid peroxidation assays. Lipid peroxidation was evaluated using the thiobarbituric acid reactive substances (TBAR) assay. Lysates, normalized to 30 μg protein/sample, were analyzed using a TBAR assay kit (Cayman Chemical). Malondialdehyde (MDA), a lipid peroxidation byproduct, was quantified. Lysates were incubated with thiobarbituric acid (TBA) at 95 °C for 55 min, forming the MDA-TBA adduct, which was measured spectrophotometrically at 545 nm. MDA levels were determined by comparison to a standard curve.

GSH/GSSG detection. The GSH/GSSG ratio was quantified using a commercial kit (Thermo-Fisher Scientific Invitrogen). Lysates were incubated with DTNB, glutathione reductase, and NADPH. The reaction mixture was added to the lysates, and absorbance at 425 nm was monitored spectrophotometrically. GSH and GSSG concentrations were determined by interpolation from a standard curve. The GSH/GSSG ratio was normalized to protein content.

### Constitutively-active mutant Akt1

NSCLC cells were transduced with the lentivirus expressing the constitutively-active Akt1 (caAkt1, S473D) provided by Dr. Cao [36], and this process lasted for 48 h. Subsequently, puromycin was introduced for an additional 96 h to create stable NSCLC cells, with the presence of caAkt1 being verified through Western blotting analysis.

### Akt1/2 shRNA

Lentiviral particles containing Akt1/2 shRNA, also supplied by Dr. Cao [36], were introduced to the cultured NSCLC cells. After a 48 h incubation, the cells were maintained in a puromycin-supplemented medium for another 96 h. The efficiency of Akt1/2 silencing was routinely through verified via Western blotting analysis.

### Testing other cellular functions and gene/protein expression

Identical quantities of NSCLC cells and normal lung epithelial cells, subjected to specified genetic modifications or treatments, were cultured under optimized conditions for predetermined durations. A comprehensive panel of assays were employed to characterize cellular functions, including cell viability (CCK-8), proliferation (nuclear EdU/DAPI staining), colony formation (clonogenicity), migration (“Transwell” assay), invasion (“Matrigel Transwell” assay), apoptosis (caspase-3 activity, nuclear TUNEL/Hoechst 33342 staining, Annexin V-PI flow cytometry), cytosol cytochrome C ELISA, necrosis (trypan blue exclusion) [26, 34, 35]. Detailed methodologies for these assays have been previously published [26, 34, 35]. Gene expression analysis (qPCR) and protein expression profiling (Western blotting) were performed as described in our prior studies [34, 35]. Uncropped Western blotting images are provided in Supplementary Figure S1.

### Xenograft studies

The detailed protocols were described early [27]. Immunodeficient nude mice (5-6 weeks old, 18.1-19.1 g in weights) were purchased from SLAC Laboratory Animal Co., Ltd. (Shanghai, China) and were housed in a specific pathogen-free (SPF) environment at the Animal Facility of Nanchang University. Equal numbers of male and female mice were employed in this study. Subcutaneous xenograft tumors were established by injecting five million pNSCLC-1 cells into the flanks of each mouse. Tumor growth was monitored, and animals were randomized into two experimental groups when tumors reached an average volume of 100 mm³. Experimental groups received intratumoral injections of the indicated adeno-associated virus (AAV) vector (2.5 μL per tumor, 1.0 × 10^9^ PFU). Tumor dimensions were measured to calculate tumor volume using standard formulas. All animal experiment procedures were performed in accordance with the Animal Ethics Committee of Nanchang University (CDYFY-IACUC-202406QR026).

### Immunohistochemistry (IHC) and tissue fluorescence staining

Tumor specimens were formalin-fixed, paraffin-embedded, and sectioned into 4 μm thick slices. Deparaffinization and rehydration were performed using standard protocols, followed by antigen retrieval in citrate buffer. Nonspecific binding was blocked. The slides were subjected to the nuclear Ki-67 staining based on the attached protocols from Biyuntian (Wuxi, China). Alternatively, xenograft sections underwent terminal deoxynucleotidyl transferase dUTP nick end labeling (TUNEL) staining using a commercial kit (Biyuntian) to detect apoptotic cells. Nuclear counterstaining with DAPI and fluorescence microscopy enabled visualization of stained tissue cells.

Statistical analysis. To minimize experimental bias, investigators were blinded to group allocations throughout the in vitro study. All experiments were independently replicated five times, yielding consistent results. One-way analysis of variance (ANOVA) followed by Tukey’s multiple comparison test was employed for comparisons involving multiple groups. For pairwise comparisons, Student’s t-test was utilized. Data were presented as mean ± standard deviation (SD), and statistical significance was defined as a *p*-value < 0.05.

## Results

### Elevated *TIMM23* expression correlates with unfavorable clinical parameters in NSCLC

**Fig. 1:**
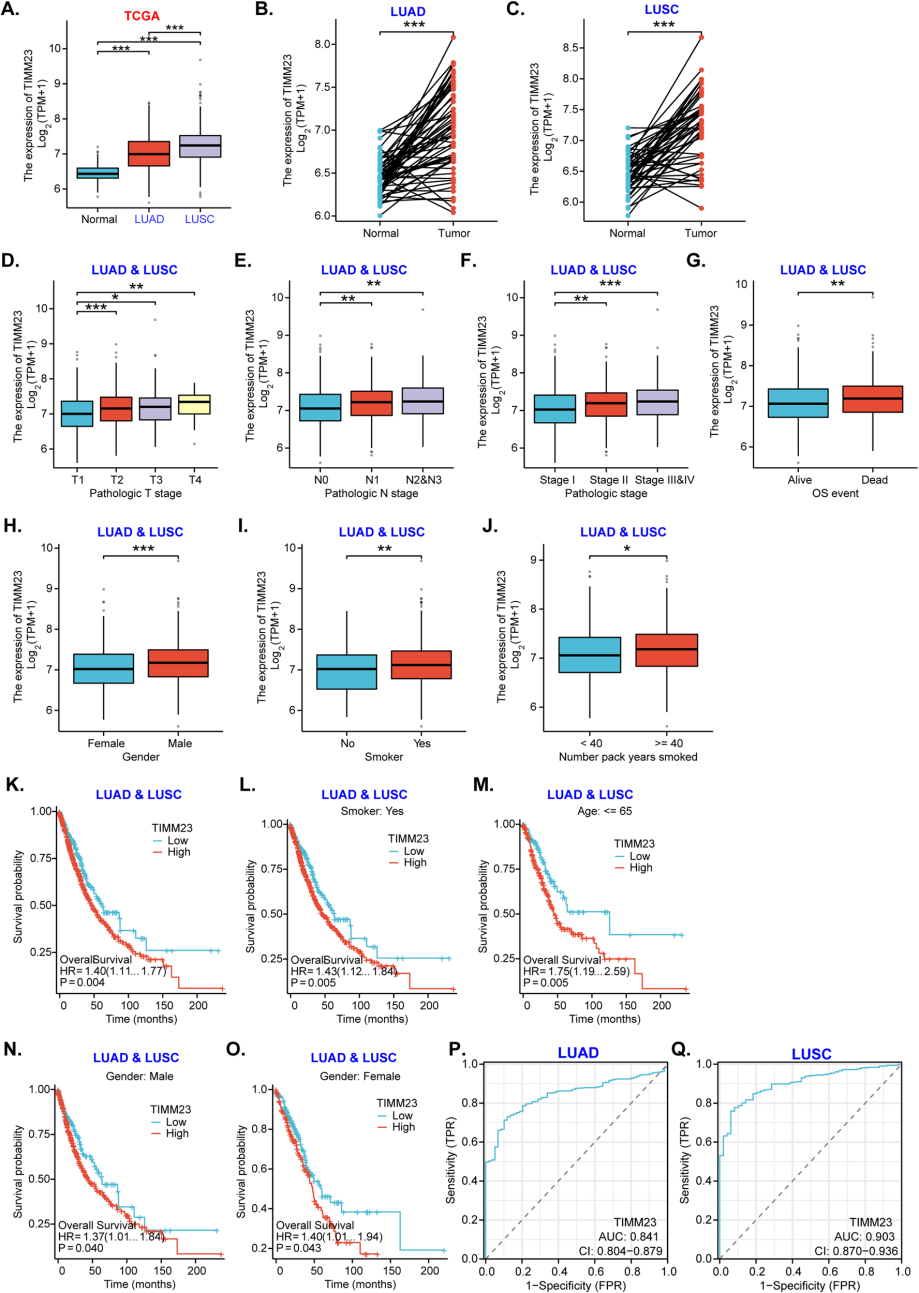
Elevated *TIMM23* expression correlates with unfavorable clinical parameters in NSCLC. The Cancer Genome Atlas (TCGA) dataset shows *TIMM23* mRNA transcript levels in lung adenocarcinoma (LUAD) tissues, lung squamous cell carcinoma (LUSC) tissues or normal lung tissues (“Normal”) (**A**). TCGA dataset shows *TIMM23* mRNA transcript levels in LUAD/LUSC tissues or paired surrounding normal tissues (**B** and **C**). Subgroup analyses shows *TIMM23* expression in LUAD&LUSC tissues in the described patients (**D**-**J**). Kaplan-Meier survival curves showing the relationship between *TIMM23* expression and overall survival of the described LUAD&LUSC patients (**K–O**). The receiver operating characteristic (ROC) curves assessing *TIMM23* expression for its predictive value in LUAD or LUSC patients (**P** and **Q**). TPM = transcripts per million, AUC = area under the curve, CI = confidence interval, HR = hazard ratio, TPR = true positive rate, FPR = false positive rate, OS = overall survival. * indicates *P* < 0.05, ** indicates *P* < 0.01, *** indicates *P* < 0.001.

First, The Cancer Genome Atlas (TCGA) datasets shows *TIMM23* expression levels in normal lung tissues, lung adenocarcinoma (LUAD) tissues, and lung squamous cell carcinoma (LUSC) tissues. The findings revealed a significant upregulation of *TIMM23* expression in both LUAD and LUSC compared to normal lung tissue (Fig. 1A). Furthermore, paired tissue analyses demonstrated higher *TIMM23* expression in LUAD or LUSC tissues compared to their corresponding normal tissues (Fig. 1B and C). We further demonstrated a positive correlation between *TIMM23* expression and tumor stage, with higher expression levels observed in advanced-stage LUAD & LUSC tumors, including T3/T4 stage tumors (Fig. 1D), N2/N3 tumors (Fig. 1E), and Stage III-IV tumors (Fig. 1F). Additionally, *TIMM23* expression was significantly higher in LUAD&LUSC tumors from deceased patients compared to those from surviving patients (Fig. 1G).

Further analyses revealed significant differences in *TIMM23* expression based on gender (Fig. 1H), with higher expression observed in male LUAD & LUSC patients (Fig. 1H). Moreover, smoking status and the number of pack years smoked were positively associated with *TIMM23* expression in LUAD & LUSC patients (Fig. 1I and J). Kaplan-Meier survival analysis demonstrated that LUAD&LUSC patients with high *TIMM23* expression had significantly poorer overall survival compared to those with low expression (Fig. 1K). This association was evident in patients with a smoking history (Fig. 1L) and those younger than 65 (Fig. 1M). Gender-specific analysis revealed that high *TIMM23* expression was associated with worse survival outcomes in both male and female LUAD & LUSC patients (Fig. 1N and O). Receiver operating characteristic (ROC) curve analysis indicated that TIMM23 had excellent diagnostic efficiency in differentiating between cancer and normal tissues. The area under the curve (AUC) was 0.841 for LUAD patients (Fig. 1P) and 0.903 for LUSC patients (Fig. 1Q), suggesting its potential as a diagnostic biomarker.

### Single-cell RNA sequencing shows TIMM23 overexpression in NSCLC cells

**Fig. 2:**
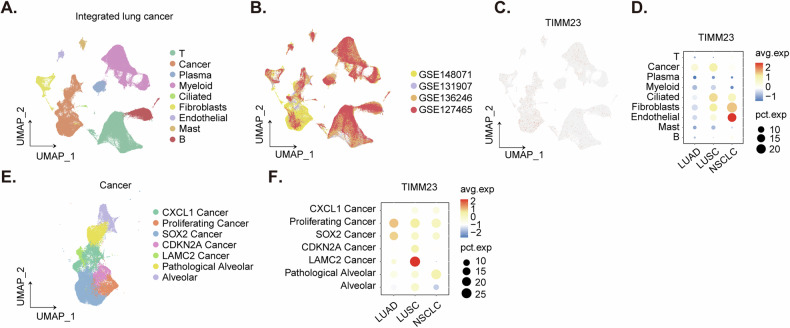
Single-cell RNA sequencing shows TIMM23 overexpression in NSCLC cells. Dimensionality reduction plot illustrating cell type annotations in the integrated lung cancer scRNA-seq dataset (**A**). Dimensionality reduction plot showing the origin of integrated data in the lung cancer scRNA-seq dataset (**B**). Dot plot demonstrating *TIMM23* expression across different cell types in the lung cancer scRNA-seq dataset (**C**). Expression density plot depicting the distribution of *TIMM23* expression levels within various cell types in the lung cancer scRNA-seq dataset (**D**). Subclustering analysis of cancer cell clusters in the lung cancer scRNA-seq dataset (**E**). Expression of *TIMM23* in different cancer cell subclusters (**F**).

A comprehensive analysis of integrated lung cancer single-cell RNA sequencing (scRNA) data revealed a distinct expression pattern of *TIMM23* across various cell types. Cell type annotations were provided by the original authors [37], allowing for a detailed examination of *TIMM23* expression within specific cell populations. Dimensionality reduction techniques were employed to visualize cell annotations (Fig. 2A) and the origin of integrated data (Fig. 2B). Dot plots (Fig. 2C) and expression density plots (Fig. 2D) demonstrated a preferential expression of TIMM23 in cancer cells, fibroblasts, and endothelial cells. Within the cancer cell cluster, *TIMM23* expression was significantly elevated compared to normal lung tissue (Fig. 2C and D). To further investigate the role of *TIMM23* in cancer cell heterogeneity, cancer cell clusters were extracted and subclustered (Fig. 2E-F). These analyses revealed a particularly high expression of *TIMM23* within the proliferating cancer cell subcluster, suggesting a potential association between *TIMM23* and cancer progression (Fig. 2E-F).Overall, these findings highlight the selective expression of TIMM23 in cancer cells of NSCLC mass and provide insights into its potential role in tumorigenesis.

### Overexpression of TIMM23 in both NSCLC tumor tissues and various NSCLC cells

**Fig. 3:**
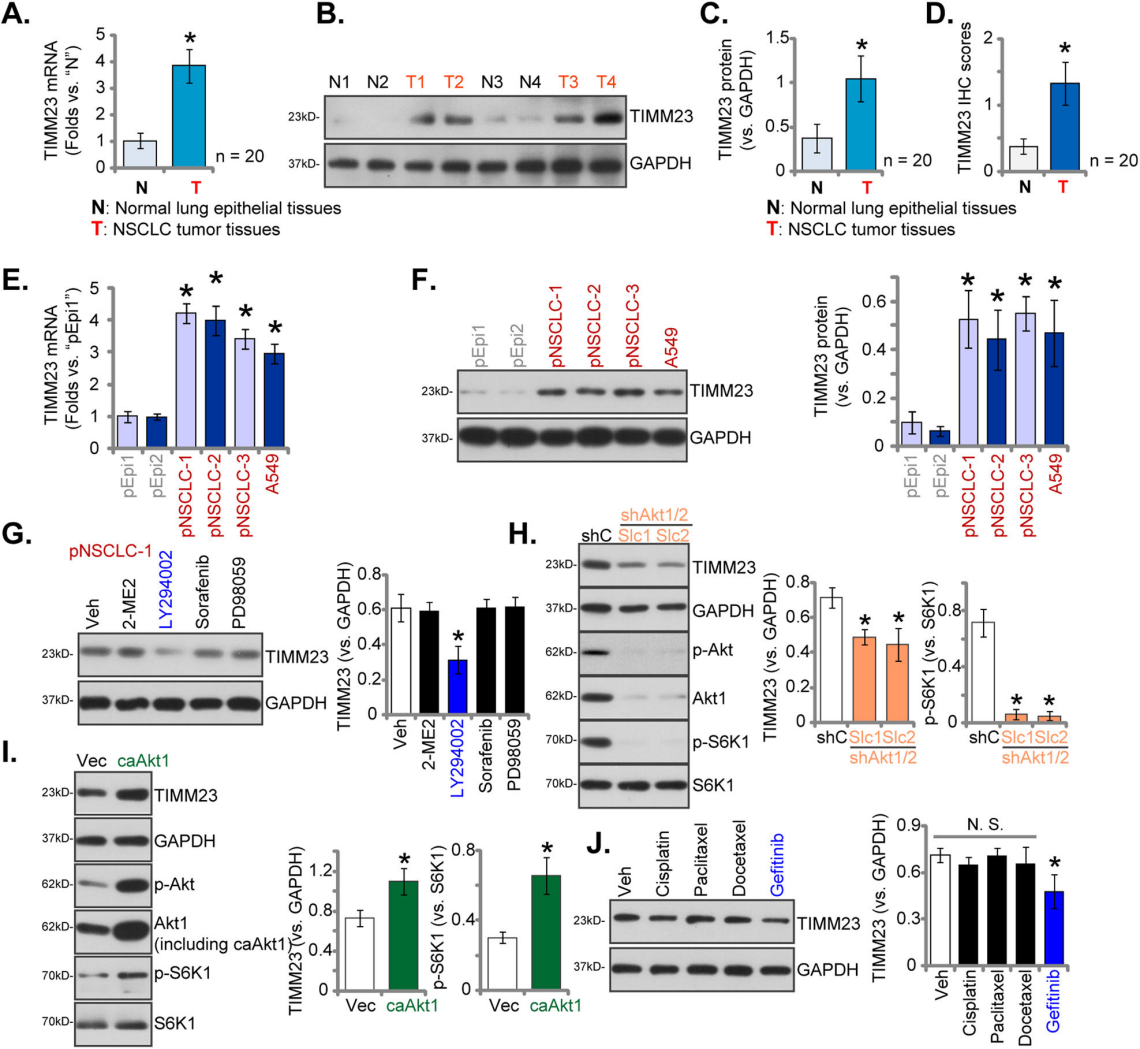
Overexpression of TIMM23 in both NSCLC tumor tissues and various NSCLC cells. Expression patterns of *TIMM23* mRNA and protein were evaluated in tumor (“T”) and adjacent normal lung epithelial (“N”) tissues from twenty (N = 20) primary NSCLC patients (**A**-**D**). *TIMM23* mRNA and protein levels were subsequently assessed in a panel of primary and immortalized NSCLC cells and primary human lung epithelial cells (**E** and **F**). The primary pNSCLC-1 cells were treated with Sorafenib (5 µM), PD98059 (5 µM), LY294002 (5 µM), 2-Methoxyestradiol (2ME) (10 µM) or vehicle control (Veh) for 12 h, MTCH2 and GAPDH protein expression was tested (**G**). Expression levels of listed proteins in pNSCLC1 cells with a constitutively active Akt1 mutant (S473D, caAkt1), the empty vector (Vec), Akt1/2 shRNA-expressing lentiviral construct (shAkt1/2) or the scramble shRNA lentiviral construct (shC) were shown (**H** and **I**). pNSCLC-1 cells were treated with Docetaxel (5 µM), Paclitaxel (100 nM), Cisplatin (5 µM), Gefitinib (0.5 µM) or vehicle control (Veh) for 12 h, MTCH2 and GAPDH protein expression was tested (**J**). Quantitative data are expressed as mean ± standard deviation (SD). Statistical significance was tested relative to “N” tissues, “pEpi1” cells, “Veh”, “shC” or “Vec” (*P < 0.05), with non-significant differences denoted as “N.S.” (*P* > 0.05).

To assess TIMM23 expression within localized NSCLC, tumor (“T”) and paired adjacent normal lung epithelial (“N”) tissues were procured from twenty (n = 20) primary NSCLC patients (LUAD, stage III-IV). Quantitative PCR (qPCR) analysis revealed a substantial elevation in *TIMM23* mRNA levels within NSCLC tumor tissues relative to corresponding normal lung epithelia (Fig. 3A). Western Blotting analysis of lysates from four representative NSCLC tumors (“T1”-“T4”) corroborated these findings, demonstrating augmented TIMM23 protein expression (Fig. 3B). Collective analysis of all twenty tissue specimens confirmed a statistically significant upregulation of TIMM23 protein within NSCLC tumors (Fig. 3C). Quantitative analysis of IHC scores across all twenty tissue samples yielded a statistically significant increase in TIMM23 protein IHC staining within the NSCLC tumors (Fig. 3D).

To further elucidate TIMM23 expression patterns, a comparative analysis was undertaken across diverse NSCLC cells, including primary human NSCLC cells (pNSCLC-1/2/3) [26, 34, 35] and the immortalized A549 cell line. qPCR demonstrated a marked upregulation of *TIMM23* mRNA in both primary and immortalized NSCLC cells relative to primary human lung epithelial cells (“pEpi1” and “pEpi2”, [26, 34, 35]) (Fig. 3E). Western blotting further showed elevated TIMM23 protein levels in NSCLC cells compared to the significantly lower expression observed in lung epithelial cells (Fig. 3F). These data collectively reinforce the notion of TIMM23 upregulation in NSCLC.

To elucidate the potential upstream mechanisms governing MTCH2 expression in NSCLC cells, we employed a range of pharmacological inhibitors. In pNSCLC-1 cells, treatment with the Raf kinase inhibitor Sorafenib [38] or the Erk-MAPK inhibitor PD98059 did not significantly alter MTCH2 protein levels (Fig. 3G). Similarly, 2-Methoxyestradiol (2ME), a compound known to inhibit HIF-1α protein synthesis, nuclear translocation, and transcriptional activity [39, 40], also failed to affect MTCH2 expression (Fig. 3G). In contrast, the application of LY294002, a potent inhibitor of the PI3K-Akt signaling pathway [41], resulted in a marked downregulation of MTCH2 protein expression in pNSCLC-1 cells (Fig. 3G). These findings suggest that the PI3K-Akt signaling cascade may play an important role in the regulation of MTCH2 expression in NSCLC.

To further validate this hypothesis, we introduced a lentivirus encoding Akt1/2 shRNA, generously provided by Dr. Cao [36], into pNSCLC-1 cells, resulting in the establishment of two stable cell selection: shAkt1/2-Slc1 and shAkt1/2-Slc2 (Fig. 3H). Analysis of these cells demonstrated a significant reduction in Akt1 expression, accompanied by decreased phosphorylation of Akt (Ser-473) and S6K1 (Thr-389) (Fig. 3H). This downregulation was paralleled by a corresponding decrease in MTCH2 protein expression (Fig. 3H). Conversely, the introduction of a constitutively active Akt1 mutant (S473D, caAkt1), also provided by Dr. Cao [36], led to an increase in the phosphorylation of both Akt and S6K1 (Fig. 3I), which was associated with elevated MTCH2 protein levels in pNSCLC-1 cells (Fig. 3I). Collectively, these results underscore the involvement of the PI3K-Akt signaling pathway in the modulation of MTCH2 expression in NSCLC cells.

We also evaluated the effects of several drugs commonly employed in the treatment of NSCLC on TIMM23 expression, specifically Docetaxel, Paclitaxel, Cisplatin, and Gefitinib [5, 42]. Notably, among the agents assessed, only the epidermal growth factor receptor (EGFR) inhibitor Gefitinib elicited a moderate downregulation of TIMM23 protein levels in pNSCLC-1 primary cells (Fig. 3J), while the other agents demonstrated no significant effect (Fig. 3J).

### Identification and functional characterization of TIMM23-associated genes

**Fig. 4:**
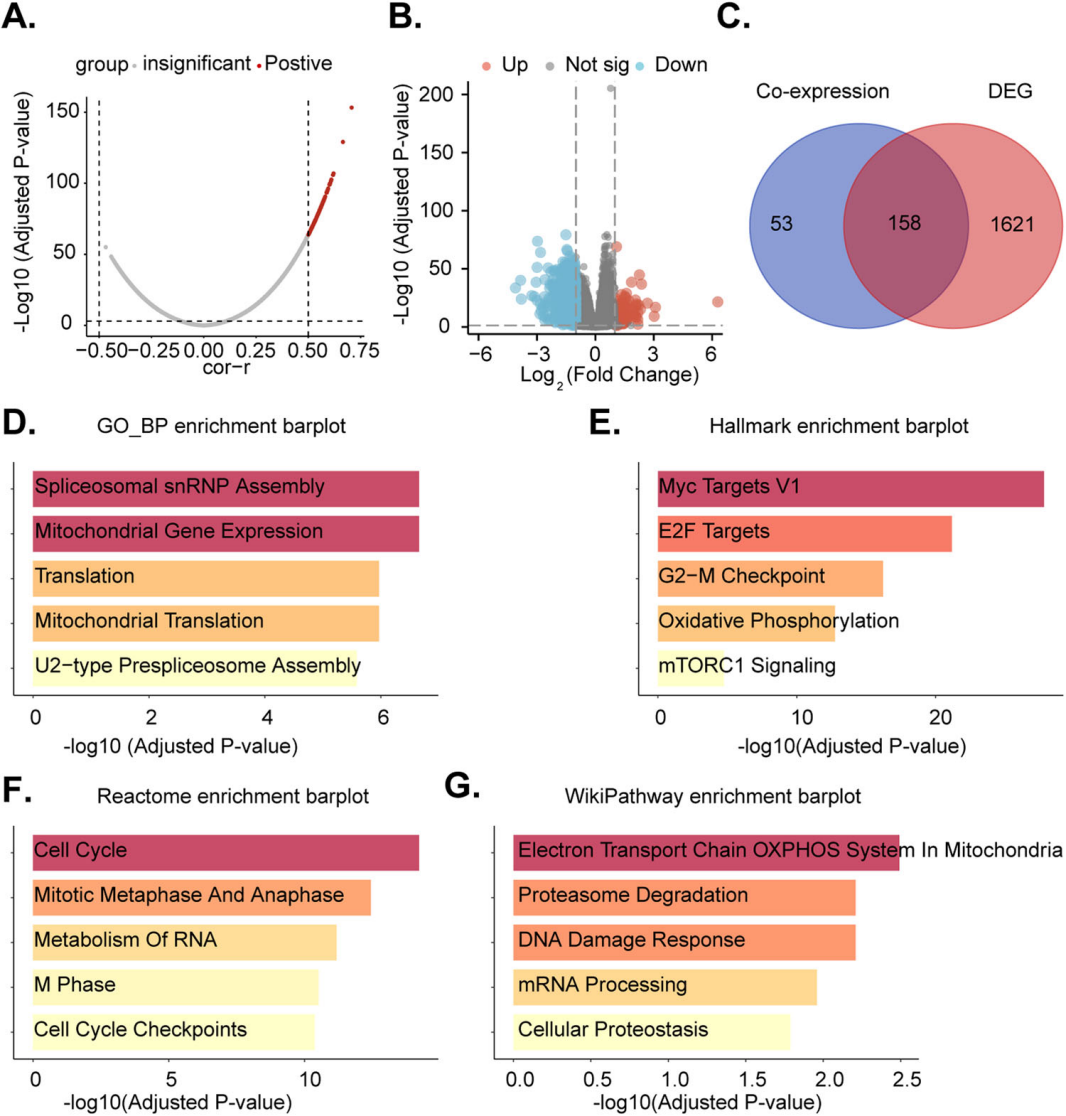
Identification and functional characterization of TIMM23-associated genes. Volcano plot depicting co-expressed genes (CEGs) with TIMM23 in TCGA-LUAD and TCGA-LUSC datasets (**A**). Boxplot differentially expressed genes (DEGs) identified between high and low TIMM23 expression groups in TCGA-LUAD and TCGA-LUSC datasets (**B**). Venn diagram showing the intersection of co-expressed and differentially expressed genes, resulting in a list of 158 genes (**C**). Enrichment analysis plots depicting the enrichment of the 158 genes in Gene Ontology Biological Processes (GO_BP), Hallmark gene sets, Reactome pathways, and WikiPathway pathways (**D**-**G**).

Utilizing TCGA-LUAD and TCGA-LUSC lung cancer datasets, a correlation analysis of TIMM23 was conducted. Employing a stringent threshold of *P* < 0.05 and a correlation coefficient *R* > 0.5, 211 co-expressed genes were identified (Fig. 4A). Subsequently, patients were dichotomized into high and low TIMM23 expression groups based on the median expression (Fig. 4B). Differential gene expression analysis revealed 1779 genes with an adjusted *P*-value < 0.05 and a fold change > 1.5 (Fig. 4B). The intersection of these two gene sets yielded a list of 158 genes (Fig. 4C). The GO_BP enrichment plot reveals a significant overrepresentation of genes involved in core cellular processes, including gene expression, splicing, and mitochondrial function (Fig. 4D). The Hallmark enrichment plot highlights the involvement of the 158 genes in cancer-related hallmark gene sets. Enrichment of terms include “Myc Targets V1,” “E2F Targets,” and “G2-M checkpoint” as well as “oxidative phosphorylation” and “mTORC1 signaling” (Fig. 4E). The Reactome enrichment plot further supports the involvement of the 158 genes in cell cycle regulation. Terms such as “cell cycle,” “mitotic metaphase and anaphase,” and “M phase” were highly enriched (Fig. 4F). The WikiPathway enrichment plot broadens the perspective by highlighting the involvement of the 158 genes in various cellular processes (Fig. 4G). Overall, the enrichment analysis results collectively suggest that the 158 genes are significantly enriched for genes involved in fundamental cellular processes, particularly those related to OXPHOS and mitochondrial function.

### TIMM23 silencing compromises mitochondrial function in NSCLC cells

**Fig. 5:**
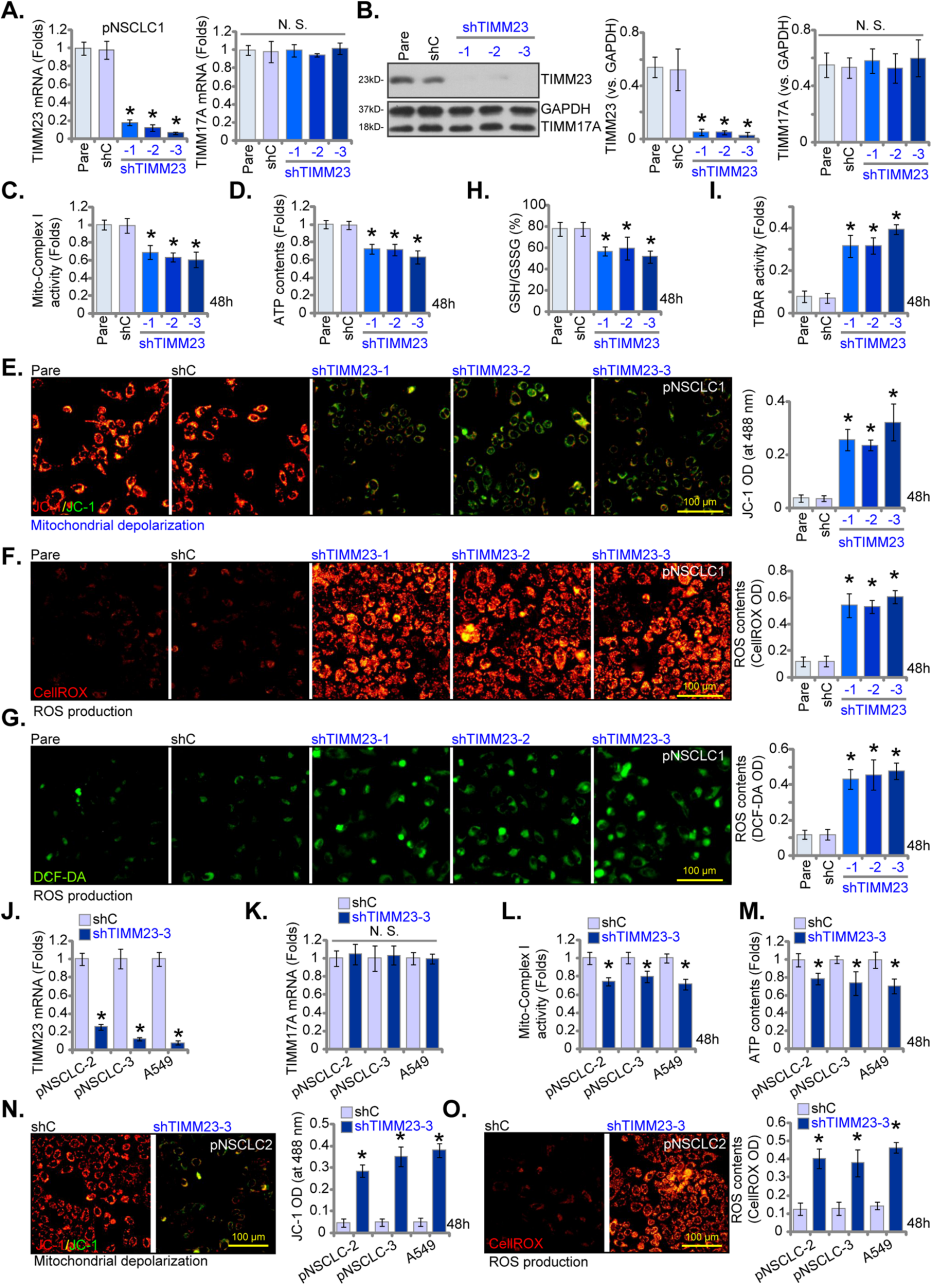
TIMM23 silencing compromises mitochondrial function in NSCLC cells. Primary pNSCLC-1 cells were subjected to individual treatments with three distinct TIMM23-specific shRNAs (shTIMM23-1, shTIMM23-2, and shTIMM23-3) or a control scramble shRNA (shC). Subsequent to treatment, mRNA and protein expression levels of both TIMM23 and TIMM17A were measured (**A** and **B**). The cells were cultivated for designated time periods (48 h), the comprehensive mitochondrial functional characterization was conducted, including assessment of mitochondrial complex I activity (**C**), cellular ATP content (**D**), mitochondrial depolarization (JC-1 monomer accumulation, **E**) and ROS production (CellROX and DCF-DA intensities, **F** and **G**). The GSH/GSSG ratio (**H**) and lipid peroxidation (TBAR intensity, **I**) were also tested. Additionally, stable cells expressing either shC or shTIMM23-3 were generated from other primary NSCLC cells (pNSCLC-2, pNSCLC-3) and the A549 immortalized cell line. mRNA expression levels of *TIMM23* and *TIMM17A* were subsequently assessed (**J** and **K**). Equal numbers of these cells were cultured for specific durations (48 h) to assess mitochondrial complex I activity (**L**), cellular ATP content (**M**), mitochondrial depolarization (JC-1 monomer accumulation, **N**) and ROS contents (CellROX intensity, **O**). “Pare” stands for the parental control cells. Quantitative data are expressed as mean ± standard deviation (SD, n = 5). Statistical significance was tested relative to “shC” control cells (*P < 0.05), with non-significant differences denoted as “N.S.” (*P* > 0.05). All experimental procedures were independently replicated five times (biological repeats), demonstrating consistent outcomes. Scale bars represent 100 µm.

To investigate the potential impact of TIMM23 silencing on mitochondrial function within NSCLC cells, we implemented a shRNA-mediated knockdown strategy. Lentiviral vectors encoding three distinct shRNAs targeting TIMM23 (“shTIMM23-1”, “shTIMM23-2”, and “shTIMM23-3”, targeting non-overlapping sequences) were introduced into pNSCLC-1 primary cells (as reported early [26, 34, 35]), resulting in the generation of stable cells following puromycin selection. Quantitative analysis revealed a significant reduction in both *TIMM23* mRNA and protein levels in these pNSCLC-1 primary cells (Fig. 5A and B), while TIMM17A expression remained unaffected (Fig. 5A and B). Concomitantly, a marked decrease in mitochondrial complex I activity was observed in shTIMM23 pNSCLC-1 cells, accompanied by a concomitant reduction in ATP levels (Fig. 5C and D).

Furthermore, TIMM23 knockdown induced mitochondrial membrane depolarization in pNSCLC-1 cells, as indicated by a shift in JC-1 fluorescence from red (aggregate form) to green (monomeric form) (Fig. 5E). A concomitant increases in both CellROX (red) and DCF-DA (green) fluorescence intensities were observed in shTIMM23 pNSCLC-1 cells, indicative of elevated reactive oxygen species (ROS) levels (Fig. 5F and G). These findings were corroborated by a significant reduction in the GSH/GSSG ratio (Fig. 5H). Moreover, TIMM23 silencing led to lipid peroxidation in pNSCLC-1 cells, as evidenced by the marked increase in TBAR intensity (Fig. 5I).

To assess the generalizability of TIMM23 knockdown across diverse NSCLC cell models, lentiviral delivery of shTIMM23-3 was employed in primary human NSCLC cells from distinct patients (pNSCLC-2 and pNSCLC-3, as reported early [26, 34, 35]) and the immortalized A549 cell line. Stable cell populations were subsequently generated through puromycin selection. Quantitative analysis revealed a consistent and significant downregulation of *TIMM23* mRNA in all treated NSCLC cells (Fig. 5J), while *TIMM17A* mRNA levels remained unaffected (Fig. 5K). In both primary (pNSCLC-2, pNSCLC-3) and immortalized (A549) NSCLC cells, TIMM23 knockdown resulted in significant reductions in mitochondrial complex I activity and ATP production (Fig. 5L and M). Additionally, shTIMM23-3 induced mitochondrial membrane depolarization, as evidenced by an accumulation of JC-1 monomer (Fig. 5N). Concurrently, an increase in ROS production was observed, as indicated by elevated CellROX red fluorescence in both primary and A549 NSCLC cells (Fig. 5O). Collectively, these data demonstrated that TIMM23 silencing compromised mitochondrial function in NSCLC cells, leading to ATP depletion, mitochondrial dysfunction, and oxidative stress.

### TIMM23 silencing inhibits NSCLC cell proliferation and migratory capacity

**Fig. 6:**
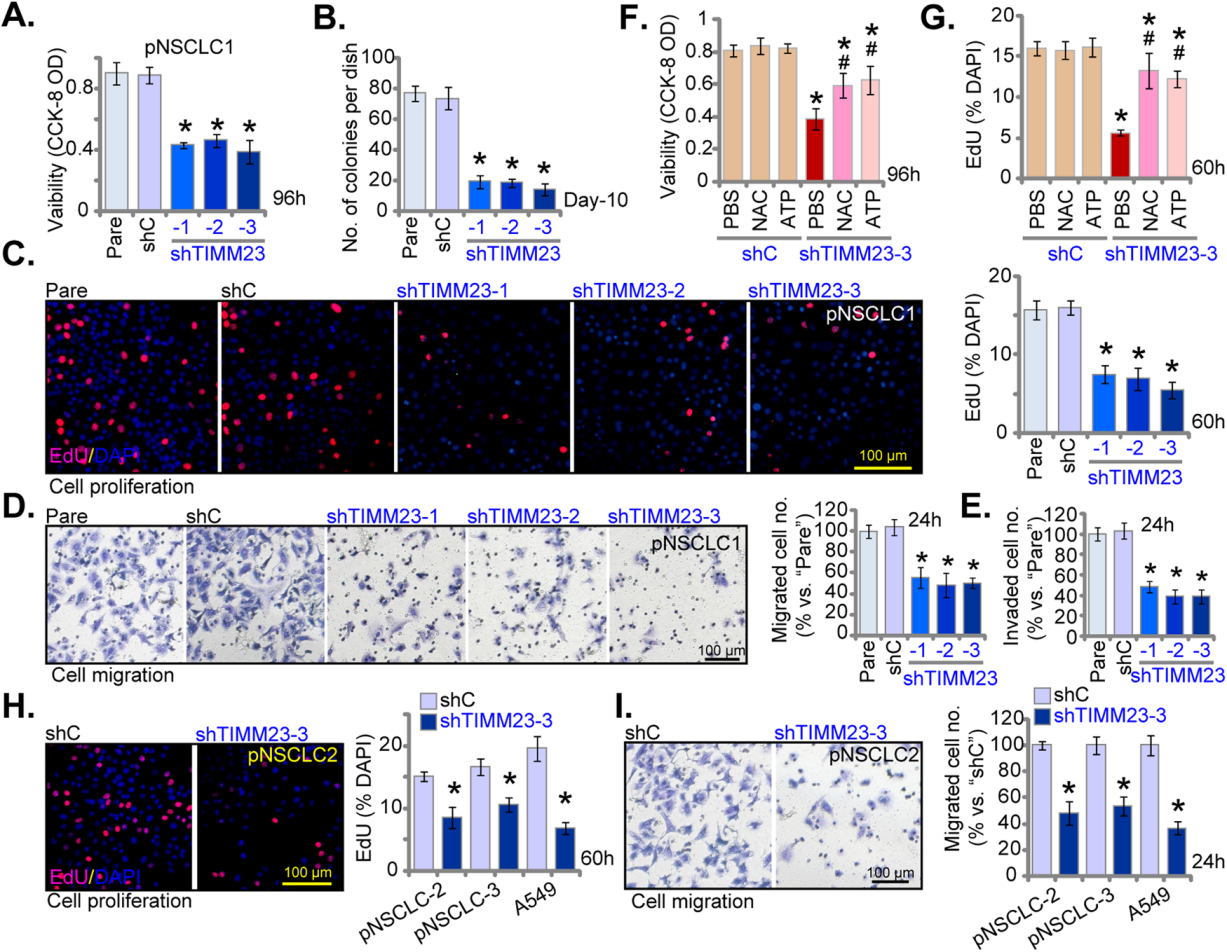
TIMM23 silencing inhibits NSCLC cell proliferation and migratory capacity. Primary pNSCLC-1 cells were subjected to individual treatments with three distinct TIMM23-specific shRNAs (shTIMM23-1, shTIMM23-2, and shTIMM23-3) or a control scramble shRNA (shC). The cells were cultivated for designated time periods, the comprehensive functional characterization was conducted, including assessment of cell viability (**A**), colony formation (**B**), cell proliferation (via testing nuclear EdU incorporation, **C**), migration (“Transwell” assays, **D**), and invasion (“Matrigel Transwell” assays, **E**). Next, pNSCLC-1 cells with shTIMM23-3 or shC were treated with either ATP (10 mM) or N-acetylcysteine (NAC, 0.5 mM) for designated time periods, the impact of these treatments on cell viability and proliferation were determined by CCK-8 (**F**) and nuclear EdU incorporation (**G**) assays, respectively. Stable cells expressing either shC or shTIMM23-3 were generated from other primary NSCLC cells (pNSCLC-2, pNSCLC-3) and the A549 cells, and cultivated for indicated time periods, cell proliferation (via testing nuclear EdU incorporation, **H**) and migration (“Transwell” assays, **I**) were tested using the same methods. “Pare” stands for the parental control cells. Quantitative data are expressed as mean ± standard deviation (SD, *n* = 5). Statistical significance was tested relative to “shC” control cells (*P < 0.05). ^#^ stands for *P* < 0.05 vs. PBS treatment (**F** and **G**). All experimental procedures were independently replicated five times (biological repeats), demonstrating consistent outcomes. Scale bars represent 100 µm.

To elucidate the functional impact of TIMM23 silencing on NSCLC cell malignancy, we again employed multiple shRNAs targeting TIMM23 (shTIMM23-1, shTIMM23-2, and shTIMM23-3, see Fig. 5). Suppression of TIMM23 expression significantly attenuated cell viability in pNSCLC-1 cells as assessed by CCK-8 assay (Fig. 6A). Moreover, TIMM23 knockdown inhibited pNSCLC-1 cell proliferation, as evidenced by reduced colony formation (Fig. 6B) and decreased nuclear EdU incorporation (Fig. 6C). TIMM23 silencing markedly impaired the migratory and invasive capacities of pNSCLC-1 cells, as determined by “Transwell” and “Matrigel invasion” assays, respectively (Fig. 6D and E). The scramble control shRNA (shC) had no appreciable effect on the cellular behaviors examined (Fig. 6A-E).

Importantly, the shTIMM23-3-mediated reduction in cell viability (Fig. 6F) and proliferation (Fig. 6G) was significantly ameliorated by exogenous cell permeable ATP supplementation or the antioxidant N-acetylcysteine (NAC), suggesting that mitochondrial dysfunction and oxidative stress may underlie the growth-inhibitory effects of TIMM23 silencing in pNSCLC-1 cells. TIMM23 knockdown using shTIMM23-3 (see Fig. 5) similarly inhibited proliferation, as assessed by suppressed EdU incorporation, and reduced cell migration in primary human NSCLC cells from independent patients (pNSCLC-2 and pNSCLC-3) and immortalized A549 cells (Fig. 6H and I). Collectively, these findings demonstrated that TIMM23 silencing inhibited NSCLC cell proliferation and migratory capacity.

### TIMM23 silencing induces apoptotic cell death in both primary and immortalized NSCLC cells

**Fig. 7:**
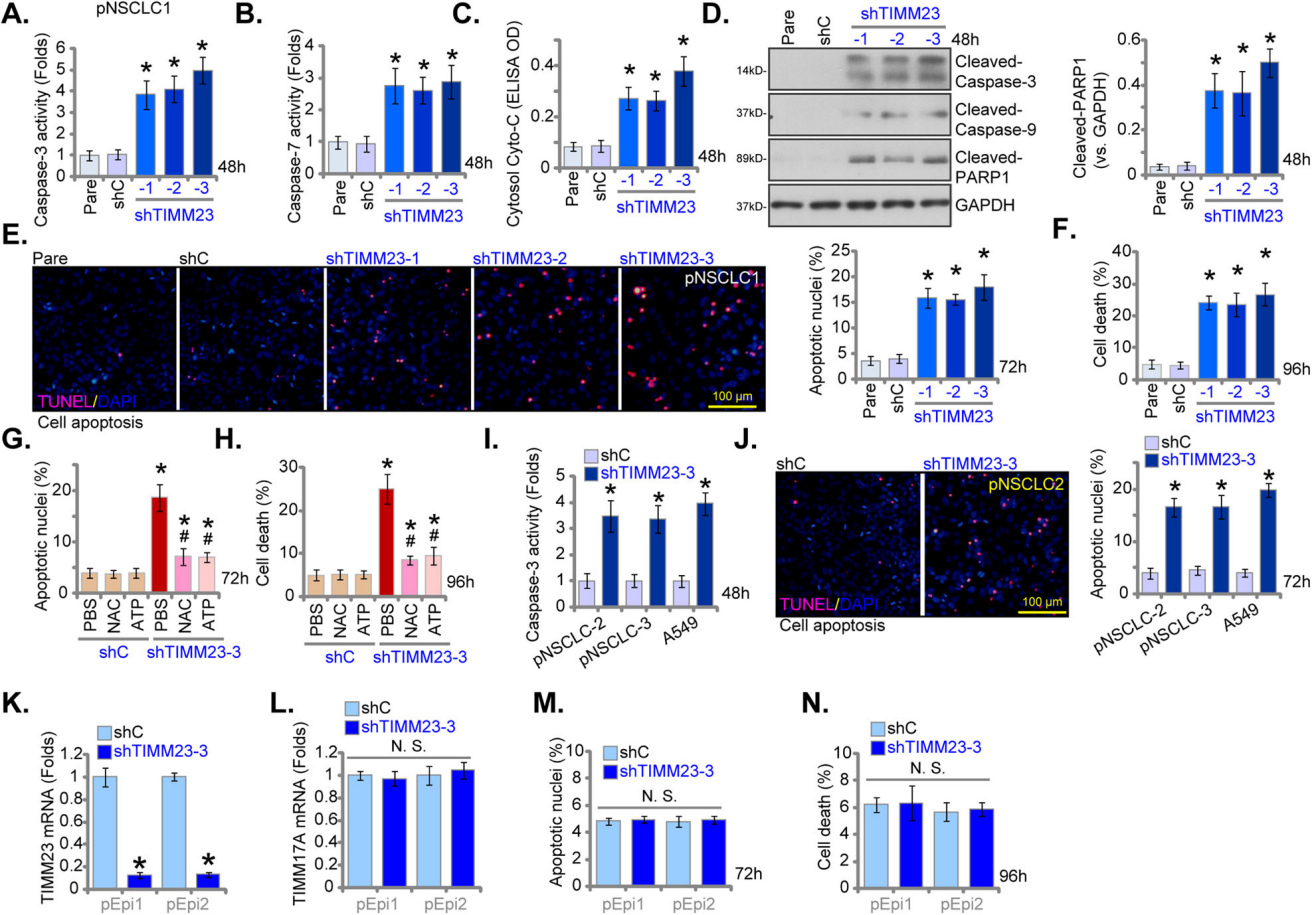
TIMM23 silencing induces apoptotic cell death in both primary and immortalized NSCLC cells. Primary pNSCLC-1 cells were subjected to individual treatments with three distinct TIMM23-specific shRNAs (shTIMM23-1, shTIMM23-2, and shTIMM23-3) or a control scramble shRNA (shC), and stable cells established after puromycin selection. The cells were cultivated for designated time periods; Apoptosis was evaluated by multiple methods, including measurement of caspase-3 (**A**) and caspase-7 (**B**) activities, detection of cytosolic cytochrome c release via ELISA (**C**), and Western blotting analysis of cleaved apoptotic proteins (**D**). Additionally, TUNEL staining (**E**) and trypan blue exclusion (**F**) assays were performed to quantify apoptosis and cell death, respectively. Next, pNSCLC-1 cells with shTIMM23-3 or shC were treated with either ATP (10 mM) or N-acetylcysteine (NAC, 0.5 mM). The impact of these treatments on apoptosis was determined by nuclear TUNEL staining (**G**) and cell death by trypan blue staining (**H**). In addition, stable cells expressing either shC or shTIMM23-3 were generated from other primary NSCLC cells (pNSCLC-2, pNSCLC-3) and the A549 immortalized cell line and cultivated for indicated time periods, the Caspase-3 activity (**I**) and cell apoptosis (nuclear TUNEL staining, **J**) were measured similarly. Alternatively, primary human lung epithelial cells (“pEpi” and “pEpi2”), with shC or shTIMM23-3, were established. mRNA expression levels of *TIMM23* and *TIMM17A* were subsequently assessed (**K** and **L**). Equal numbers of these cells were cultured for specific durations to assess cell apoptosis (via testing nuclear TUNEL incorporation, **M**) and death (via testing Trypan blue staining, **N**). “Pare” stands for the parental control cells. Quantitative data are expressed as mean ± standard deviation (SD, *n* = 5). Statistical significance was tested relative to “shC” control cells (*P < 0.05), with non-significant differences denoted as “N.S.” (*P* > 0.05). ^#^ stands for *P* < 0.05 vs. PBS treatment (**G** and **H**). All experimental procedures were independently replicated five times (biological repeats), demonstrating consistent outcomes. Scale bars represent 100 µm.

Given the observed reductions in viability, growth, and proliferation of NSCLC cells upon TIMM23 shRNA treatment, we next aimed to elucidate whether apoptosis was activated by TIMM23 silencing. As shown, pNSCLC-1 cells transfected with shTIMM23 (shTIMM23-1, shTIMM23-2, and shTIMM23-3) exhibited a significant elevation in Caspase-3 and Caspase-7 activities compared to control cells (Fig. 7A and B). Concomitantly, an increase in cytosolic cytochrome c levels was observed in TIMM23-silenced pNSCLC-1 cells (Fig. 7C). Furthermore, cleavages of Caspase-3, poly (ADP-ribose) polymerase 1 (PARP1), and Caspase-9 were induced by the applied TIMM23 shRNAs in pNSCLC-1 cells (Fig. 7D). A significant increase in TUNEL-positive nuclei was detected in pNSCLC-1 cells transfected with shTIMM23-1, shTIMM23-2, or shTIMM23-3 (Fig. 7E), indicative of apoptosis activation. Trypan blue staining revealed a marked increase in cell death in these cells (Fig. 7F).

Importantly, the apoptotic response and cell death induced by shTIMM23-3 were significantly attenuated by exogenous ATP or the antioxidant NAC (Fig. 7G and H), suggesting a potential mechanism involving mitochondrial dysfunction and oxidative stress in the apoptotic process induced by TIMM23 silencing in pNSCLC-1 cells. To investigate the potential influence of TIMM23 knockdown on additional NSCLC cells, primary pNSCLC-2 and pNSCLC-3 cells, as well as immortalized A549 cells, were subjected to shTIMM23-3 treatment (see Figs. 5 and 6). A concomitant elevation in Caspase-3 activity (Fig. 7I) and an augmented TUNEL-positive nuclear ratio (Fig. 7J) were observed. These findings indicate that TIMM23 silencing induced apoptotic cell death in both primary and immortalized NSCLC cells.

To investigate the functional role of TIMM23 silencing in primary human lung epithelial cells, pEpi1/pEpi2 cells were subjected to TIMM23 knockdown using the same shTIMM23-3 lentiviral construct (Fig. 7K). *TIMM17A* mRNA levels remained unaffected (Fig. 7L). The suppression of TIMM23 did not induce apoptosis, as assessed by nuclear TUNEL staining (Fig. 7M), nor did it significantly cause cell death (Fig. 7N) within the lung epithelial cell population. These findings indicate that TIMM23 suppression in primary human lung epithelial cells did not elicit a significant apoptotic response, supporting a cancer cell specific response by TIMM23 silencing.

### TIMM23 KO compromises mitochondrial function and suppresses NSCLC cell growth and migration

**Fig. 8:**
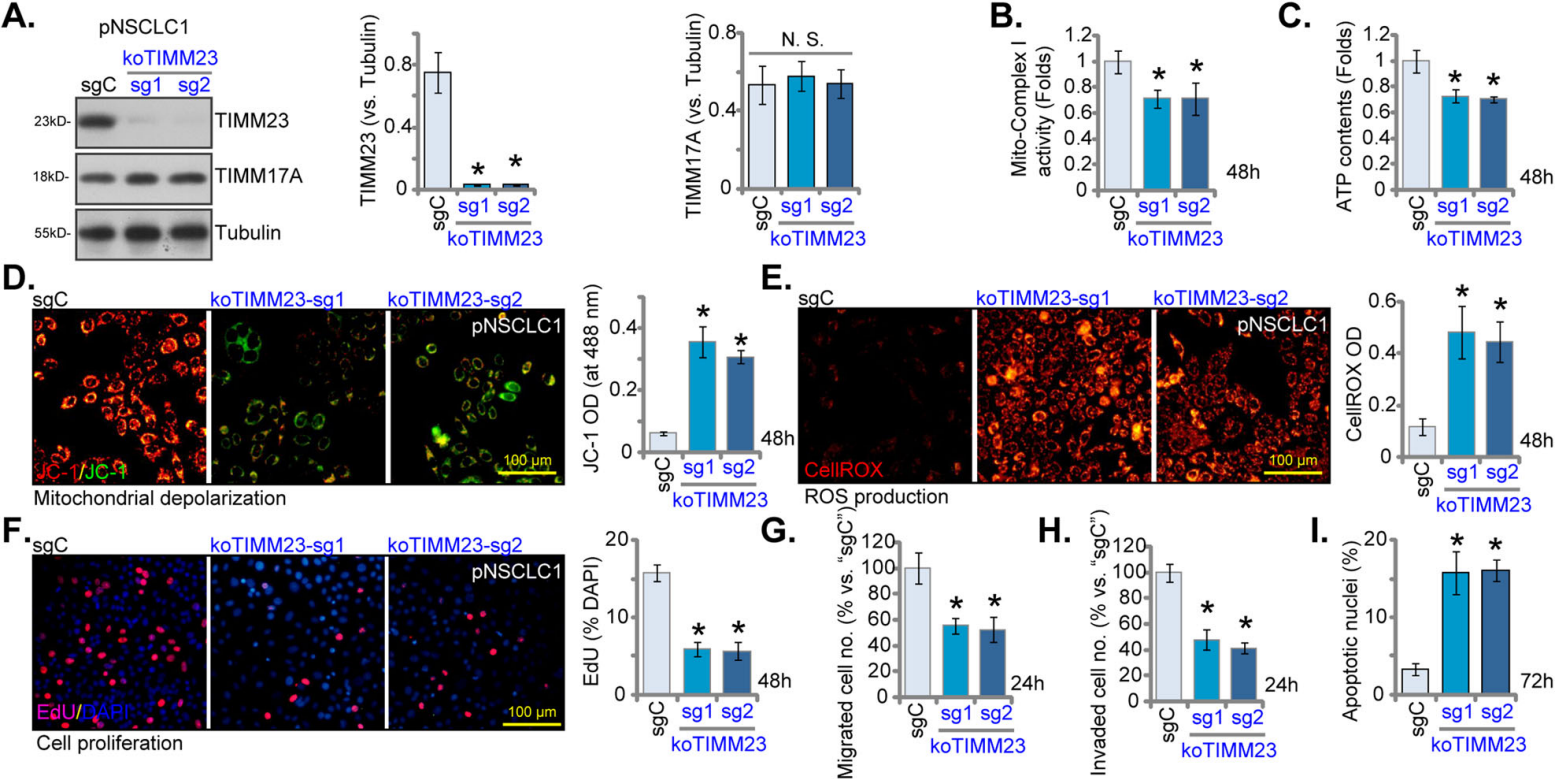
TIMM23 KO compromises mitochondrial function and suppresses NSCLC cell growth and migration. pNSCLC-1 cells harboring TIMM23 knockout (KO) alleles (“koTIMM23-sg1” and “koTIMM23-sg2”, representing two different sgRNAs) were generated through CRISPR-Cas9-mediated genome editing. Control cells (“Cas9-C”) were derived by co-transfection of the Cas9 expression construct with a non-targeting CRISPR control. TIMM23 and TIMM17A protein expression levels were assessed (**A**). The cells were cultivated for designated time periods, the comprehensive functional characterization was conducted, including assessment of mitochondrial complex I activity (**B**), cellular ATP content (**C**), mitochondrial depolarization (JC-1 monomer accumulation, **D**) and ROS production (CellROX intensity, **E**). Moreover, cell proliferation (via testing nuclear EdU incorporation, **F**), migration (“Transwell” assays, **G**), invasion (“Matrigel Transwell” assays, **H**) and apoptosis (nuclear TUNEL staining, **I**) were measured. Quantitative data are expressed as mean ± standard deviation (SD, *n* = 5). Statistical significance was tested relative to“Cas9-C” control cells (*P < 0.05), with non-significant differences denoted as “N.S.” (*P* > 0.05). All experimental procedures were independently replicated five times (biological repeats), demonstrating consistent outcomes. Scale bars represent 100 µm.

To establish TIMM23 knockout (KO) pNSCLC-1 cells, lentiviral CRISPR/Cas9 constructs with sgRNAs targeting two distinct TIMM23 sequences (“koTIMM23-sg1” and “koTIMM23-sg2”) were individually introduced. Stable KO cells were subsequently generated. TIMM23 protein levels were markedly decreased in both koTIMM23-sg1 and koTIMM23-sg2 pNSCLC-1 cells (Fig. 8A), while TIMM17A protein expression remained unaffected (Fig. 8A). Consistent with the observed effects of TIMM23 knockdown, the mitochondrial complex I activity (Fig. 8B) and ATP production (Fig. 8C) were significantly decreased in TIMM23 KO pNSCLC-1 cells. Moreover, TIMM23 KO resulted in mitochondrial depolarization, as evidenced by increased JC-1 monomer accumulation (Fig. 8D). Concurrently, an increase in CellROX intensity indicated elevated ROS production and oxidative injury within TIMM23 KO NSCLC cells (Fig. 8E). Functionally, TIMM23 KO significantly inhibited pNSCLC-1 cell proliferation and reduced EdU incorporation (Fig. 8F). Additionally, both koTIMM23-sg1 and koTIMM23-sg2 pNSCLC-1 cells exhibited impaired in vitro migration (Fig. 8G) and invasion (Fig. 8H) capacities. TIMM23 KO by the CRISPR/Cas9 method also induced apoptosis in pNSCLC-1 cells, increasing nuclear TUNEL staining (Fig. 8I). Collectively, these findings from KO experiments again highlight the critical role of TIMM23 in maintaining mitochondrial function and supporting key cellular processes within the pNSCLC-1 cells.

### Overexpression of TIMM23 enhances mitochondrial bioenergetics and promotes an aggressive NSCLC cell phenotype

**Fig. 9:**
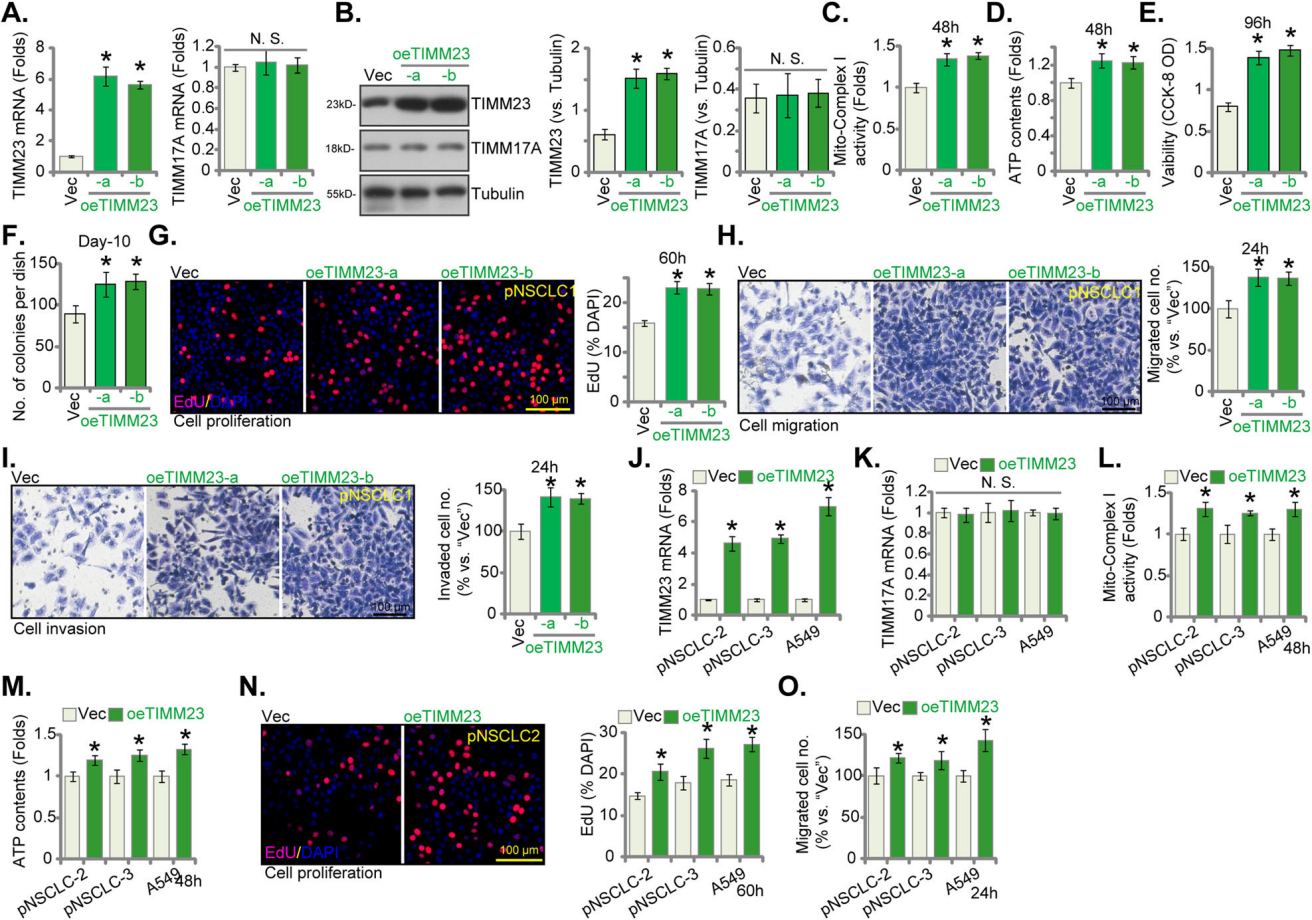
Overexpression of TIMM23 enhances mitochondrial bioenergetics and promotes an aggressive NSCLC cell phenotype. pNSCLC-1 cells stably overexpressing TIMM23 (“oeTIMM23-a” and “oeTIMM23-b”, representing two stable cell colonies) were generated, along with a vector control (“Vec”). TIMM23 and TIMM17A expression levels were examined at both the mRNA and protein levels (**A** and **B**). Cells were cultivated for designated time periods, the comprehensive functional characterization was conducted, including assessment of mitochondrial complex I activity (**C**), cellular ATP content (**D**), cell viability (CCK-8 OD, **E**), proliferation (via testing colony formation and nuclear EdU incorporation, **F** and **G**), migration (“Transwell” assays, **H**), and invasion (“Matrigel Transwell” assays, **I**). Additional NSCLC cells (pNSCLC-2, pNSCLC-3, and A549) stably expressing the same lentiviral TIMM23 construct (“oeTIMM23”) or vector control (“Vec”) were established. *TIMM23* and *TIMM17A* mRNA expression levels were determined (**J** and **K**). The cells were cultivated for designated time periods, followed by evaluation of mitochondrial complex I activity (**L**), ATP content (**M**), proliferation (**N**), and migration (**O**) similarly. Quantitative data are expressed as mean ± standard deviation (SD, *n* = 5). Statistical significance was tested relative to vector control (“Vec”) cells (*P < 0.05), with non-significant differences denoted as “N.S.” (*P* > 0.05). All experimental procedures were independently replicated five times (biological repeats), demonstrating consistent outcomes. Scale bars represent 100 µm.

The above findings establish that TIMM23 depletion, achieved through either knockdown (shRNA) or KO (CRISPR/Cas-9) strategies, exerted pronounced anti-cancer effects within both primary and immortalized NSCLC cells. We next postulated that ectopic TIMM23 overexpression would elicit contrasting outcomes. To experimentally support this hypothesis, a lentiviral vector encoding TIMM23 (“oeTIMM23”) was introduced into pNSCLC-1 cells. Subsequent puromycin selection yielded two stable cell populations, designated “oeTIMM23-a” and “oeTIMM23-b”. Compared to control vector-transduced pNSCLC-1 cells (“Vec”), oeTIMM23 pNSCLC-1 cells exhibited a marked upregulation of both *TIMM23* mRNA and protein expression (Fig. 9A and B), without concomitant alterations in TIMM17A levels (Fig. 9A and B). Significantly, ectopic TIMM23 expression led to a significant augmentation of mitochondrial function, as evidenced by increased mitochondrial complex I activity and ATP production (Fig. 9C and D). Cell viability, as measured by the CCK-8 assay, was significantly elevated in pNSCLC-1 cells overexpressing TIMM23 (Fig. 9E). Additionally, lentiviral TIMM23 overexpression promoted cell proliferation, as evidenced by augmented colony formation (Fig. 9F) and increased nuclear EdU labeling (Fig. 9G) in pNSCLC-1 cells. Furthermore, TIMM23 overexpression enhanced migratory and invasive capabilities within the pNSCLC-1 cells (Fig. 9H and I).

Subsequently, the same lentiviral vector encoding TIMM23 (oeTIMM23) was introduced into additional primary NSCLC cells (pNSCLC-2 and pNSCLC-3) and the immortalized A549 cells. Stable cell populations were generated through puromycin selection. These engineered NSCLC cells exhibited a substantial upregulation of *TIMM23* mRNA expression (Fig. 9J) without affecting the control *TIMM17A* mRNA levels (Fig. 9K). TIMM23 overexpression significantly augmented mitochondrial complex I activity (Fig. 9L) and ATP synthesis (Fig. 9M) in these primary and immortalized NSCLC cells. Furthermore, these cells demonstrated enhanced proliferation (nuclear EdU staining, Fig. 9N) and accelerated in vitro migration (Fig. 9O). Collectively, these data again underscore the critical role of TIMM23 in modulating mitochondrial function and orchestrating the malignant phenotype of NSCLC cells.

### TIMM23 silencing results in impaired mitochondrial function, suppressed proliferation, and apoptotic cell death in pNSCLC-1 xenografts

**Fig. 10:**
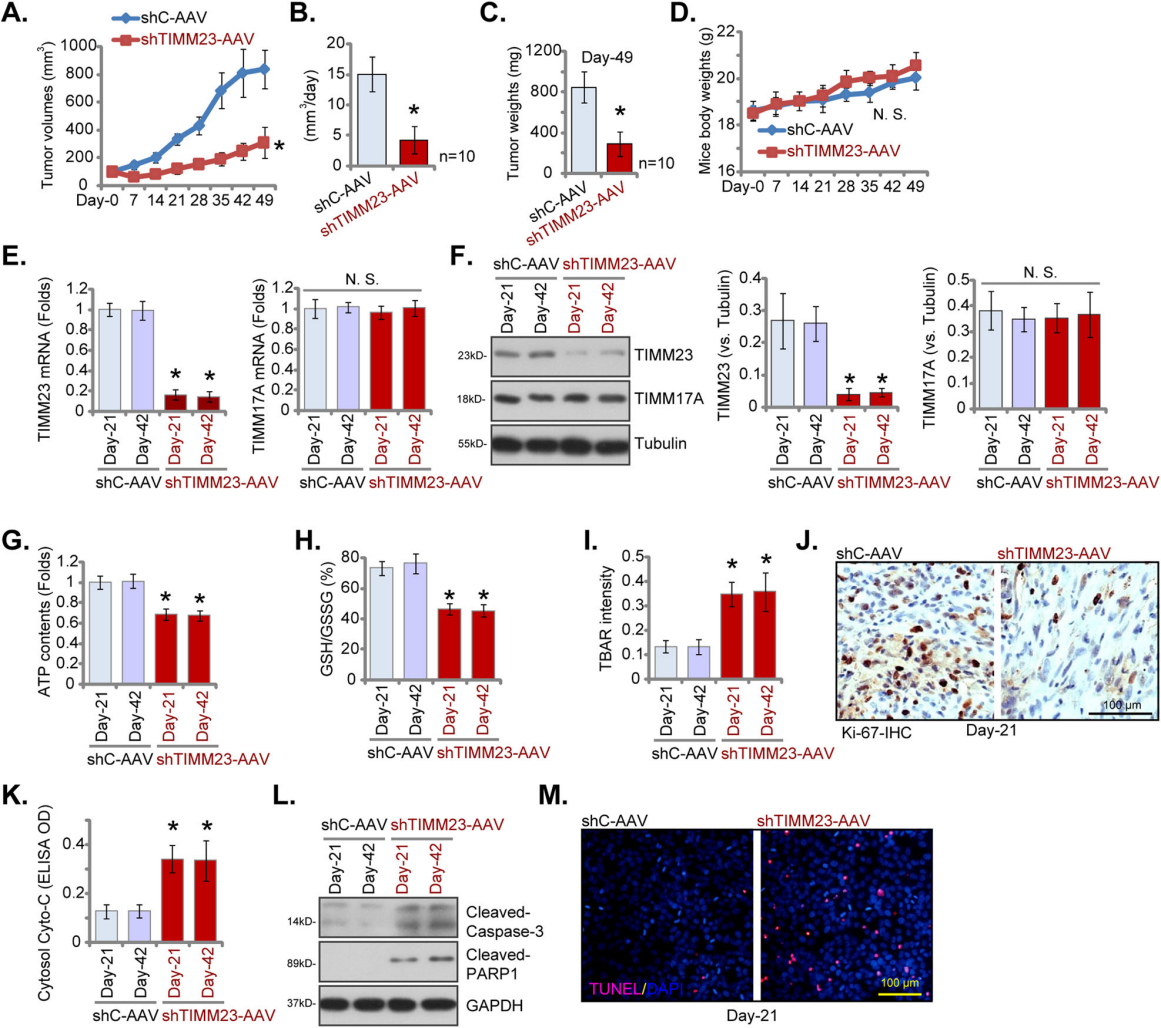
TIMM23 silencing results in impaired mitochondrial function, suppressed proliferation, and apoptotic cell death in pNSCLC-1 xenografts. Nude mice bearing pNSCLC-1 xenografts received intratumoral injections of either TIMM23 shRNA AAV (“shTIMM23-AAV”) or control shRNA AAV (“shC-AAV”) at 48-hour intervals for two consecutive rounds. Tumor volumes (**A**) and body weights of the animals (**D**) were monitored weekly. The estimated daily growth rate of pNSCLC-1 xenografts was calculated (**B**). At day 49 (“Day-49”), xenografts were excised and weighed individually (**C**). mRNA and protein expression levels were quantified in the indicated xenograft tissues (**E,** **F**, and **L**). Additionally, ATP content (**G**), the GSH/GSSH ratio (**H**), TBAR intensity (**I**) and cytosol cytochrome c content (**K**) in xenograft lysates were measured. Immunohistochemical staining was performed to evaluate nuclear Ki-67 incorporation (**J**) in the described xenograft tissue sections. TUNEL staining was conducted to detect apoptotic nuclei in the same tissue samples (**M**). Data were represented as mean ± standard deviation (SD). Statistical significance was determined by comparison to the aav-shC control group, with *P < 0.05 indicating significant differences and “N.S.” denoting non-significant differences (*P* > 0.05). Each experimental group comprised ten mice (n = 10) for panels A-D. For panels E-M, data were derived from five randomly selected tissue samples per xenograft (n = 5). Scale bars represent 100 µm.

For in vivo studies, pNSCLC-1 cells were subcutaneously inoculated into nude mice. Following a 21-day incubation period, established pNSCLC-1 xenograft tumors (designated as “Day-0”) were subjected to intratumoral administration of either TIMM23 shRNA-expressing adeno-associated virus (shTIMM23-AAV) or control shRNA AAV (shC-AAV) in respective treatment groups. Viral vector delivery was repeated at 48-hour intervals for a total of two administrations. Intratumoral injection of shTIMM23-AAV resulted in a marked inhibition of pNSCLC-1 xenograft growth (Fig. 10A). Subsequent calculation of estimated daily pNSCLC-1 xenograft growth rates, expressed as mm³ per day, corroborated the suppressive effect of shTIMM23-AAV treatment on tumor progression (Fig. 10B). At Day-49, all pNSCLC-1 xenografts were excised and individually weighed. A significant reduction in xenograft mass was observed in the shTIMM23-AAV treatment group compared to the shC-AAV control group (Fig. 10C). Notably, no significant differences in body weight were detected between the two experimental cohorts (Fig. 10D).

At 21 and 42 days post-implantation (“Day-21” and “Day-42”), one single pNSCLC-1 xenograft was carefully excised from both the shTIMM23-AAV and shC-AAV groups. Fresh tumor xenografts were divided into five sections for subsequent signaling analysis. A marked reduction in TIMM23 expression was observed in shTIMM23-AAV pNSCLC-1 xenograft tissues, as confirmed by qPCR and Western Blotting assays (Fig. 10E and F), while *TIMM17A* mRNA and protein levels remained unchanged (Fig. 10E and F). Concurrently, reduced ATP content was detected in TIMM23-deficient pNSCLC-1 xenograft tissues (Fig. 10G). Additionally, the GSH/GSSH ratio decreased following aav-shTIMM23-S1 treatment (Fig. 10H), whereas TBAR activity, indicative of lipid peroxidation, increased (Fig. 10I). A significant reduction in nuclear Ki-67 positivity was observed in pNSCLC-1 xenografts subjected to shTIMM23-AAV treatment, indicative of the in vivo anti-proliferative effect (Fig. 10J). Furthermore, cytosolic cytochrome c release was elevated in shTIMM23-AAV-treated xenograft tissues (Fig. 10K), concurrent with increased levels of cleaved-caspase-3 and cleaved-PARP-1 (Fig. 10L). The fluorescence staining of xenograft sections confirmed the induction of apoptosis in TIMM23-deficient pNSCLC-1 xenografts, as demonstrated by an augmented proportion of TUNEL-positive cells (Fig. 10M). Collectively, these findings provide compelling evidence that TIMM23 silencing resulted in impaired mitochondrial function, suppressed proliferation, and apoptosis in pNSCLC-1 xenografts.

## Discussion

Despite significant advancements in targeted therapies, the clinical management of NSCLC remains a formidable challenge [8, 10, 43]. The emergence of drug resistance and the heterogeneity of NSCLC have underscored the limitations of current therapeutic approaches [8, 10, 43]. Moreover, a substantial subset of patients harbors oncogenic alterations that remain undruggable, necessitating the identification of novel therapeutic targets to improve patient outcomes [8, 10, 43].

Mitochondria are indispensable organelles central to cellular energetics and metabolism. Their role encompasses OXPHOS for ATP generation, amino acid catabolism, macromolecular biosynthesis, fatty acid oxidation, and ionic equilibrium maintenance [19–23]. Dysfunctional mitochondrial activity is a hallmark of NSCLC and other malignancies [44]. To sustain rapid proliferation, tumor cells exhibit augmented bioenergetic demands met through increased ATP production [19–23]. Mitochondrial respiration and ATP synthesis are critical for NSCLC tumorigenesis and progression [6, 25]. Heme, an essential cofactor for OXPHOS, is overproduced and/or avidly acquired in NSCLC to support elevated mitochondrial activity. Conversely, inhibiting heme biosynthesis or uptake suppresses OXPHOS, reduces oxygen consumption, and consequently impedes NSCLC growth [6, 25].

Emerging evidence implicates aberrant overexpression of several mitochondrial proteins in NSCLC pathogenesis and progression [26, 27, 45]. Zhang et al., identified ADCK2 (AarF domain containing kinase 2), a mitochondrial kinase involved in regulating lipid metabolism and mitochondrial protein assembly, as a promising therapeutic target for NSCLC [27]. Genetic ablation or silencing of ADCK2 resulted in mitochondrial dysfunction and suppressed NSCLC cell growth [27]. Zhou et al., demonstrated the oncogenic potential of POLRMT (RNA polymerase mitochondrial) in NSCLC [45]. The authors proposed that POLRMT supports NSCLC cell growth by preserving mitochondrial DNA integrity, regulating key mitochondrial gene expression, and activating the Akt-mTOR pathway [45]. Agorreta et al., have proposed that TNF receptor-associated protein 1 (TRAP1) is a critical mitochondrial regulator influencing cell proliferation, survival, and mitochondrial function within NSCLC cells [46]. The authors established a correlation between elevated TRAP1 expression and an increased risk of disease recurrence, suggesting its potential as a prognostic biomarker and therapeutic target [46]. Our recent study elucidated the oncogenic role of YME1L (YME1 like 1 ATPase), a mitochondrial ATPase, in NSCLC cell proliferation and survival [1]. Functional impairment of mitochondria following YME1L knockdown underscored its indispensable role in mitochondrial homeostasis and NSCLC cell growth [26].

This study identifies TIMM23 as a promising prognosis marker and potential therapeutic target in NSCLC. Comprehensive bioinformatic analysis revealed a strong correlation between TIMM23 overexpression and adverse clinical outcomes in NSCLC patients. Single-cell transcriptomic data further corroborated these findings, demonstrating elevated TIMM23 expression within the NSCLC tumor cells. *TIMM23* mRNA and protein levels were significantly increased in locally-treated NSCLC tissues compared to adjacent normal lung tissue. Functional studies using shRNA-mediated knockdown and CRISPR/Cas9-mediated knockout revealed that TIMM23 is essential for cell viability, proliferation, and migration in various primary and immortalized NSCLC cell. Conversely, ectopic TIMM23 overexpression promoted these malignant phenotypes in primary NSCLC cells. In vivo, silencing of TIMM23 using target shRNA significantly suppressed tumor growth in a xenograft mouse model, underscoring its critical role in NSCLC pathogenesis.

Previous investigations have unveiled that perturbation of critical mitochondrial proteins, including POLRMT, TRAP1, ADCK2,and YME1L, through inhibition, silencing, or knockout strategies, induced mitochondrial impairment in NSCLC cells. These functional deficits manifested as diminished ATP synthesis, elevated ROS generation, oxidative injury, DNA/lipid damage, and apoptotic cell death [26, 27, 45, 46]. In contrast, ectopic augmentation of these mitochondrial factors has been shown to enhance mitochondrial bioenergetics and stimulate NSCLC cell proliferation [26, 27, 45].

Our findings underscore the critical role of TIMM23 in sustaining hyperactive mitochondrial function within NSCLC cells. Suppression of TIMM23 via genetic silencing or knockdown caused pronounced mitochondrial dysfunction, manifested by attenuated complex I activity, ATP depletion, mitochondrial membrane potential collapse, oxidative stress, and lipid peroxidation. Conversely, ectopic TIMM23 overexpression in primary human NSCLC cells augmented mitochondrial OXPHOS and ATP generation. Importantly, exogenously-added ATP or NAC treatment potently alleviated TIMM23 silencing-induced anti-NSCLC cell activity. In vivo, TIMM23 silencing within NSCLC xenografts resulted in impaired mitochondrial bioenergetics, as evidenced by diminished complex I activity, ATP levels, and lipid peroxidation.

While the subcutaneous injection of NSCLC cells followed by intratumoral administration of TIMM23 shRNA-expressing adeno-associated virus (shTIMM23-AAV) provided a valuable platform for assessing the effects of TIMM23 on NSCLC cell growth in vivo, it is crucial to acknowledge that this model may not fully recapitulate the complex in vivo environment of lung cancer. NSCLCs within the lung are subject to distinct anatomical and physiological factors, including specific immune cell interactions within the pulmonary microenvironment, the influence of pulmonary circulation dynamics, and the unique characteristics of the lung parenchyma 4, 5. These factors can significantly influence tumor behavior, therapeutic responses, and overall disease progression 4, 5. Therefore, while our findings demonstrate promising anti-NSCLC effects of shTIMM23-AAV in this model, further investigation in more physiologically relevant models, such as orthotopic lung cancer models, is warranted to confirm these findings and assess their clinical translatability.

Our findings indicate that the inhibition or silencing of Akt resulted in a downregulation of MTCH2 protein expression in pNSCLC-1 cells, whereas the expression of MTCH2 was upregulated by caAkt1. This underscores the pivotal role of the PI3K-Akt signaling pathway in the regulation of MTCH2 expression in NSCLC cells. Nonetheless, the mechanisms underlying this regulatory effect, as well as the potential involvement of additional pathways, are yet to be elucidated and will be the focus of our subsequent research endeavors.

Collectively, these data establish TIMM23 as a key determinant of mitochondrial hyperfunction and a pivotal driver of NSCLC tumorigenesis.

## Supplementary information

Original data

Figure S1.

## Data Availability

All data are available upon request.
