# Supplementary figures and images for: TIMM23 overexpression drives NSCLC cell growth and survival by enhancing mitochondrial function
Source: Cell Death Dis. 2025 Mar 13;16(1):174. doi: 10.1038/s41419-025-07505-3 (PMC11906786; doi:10.1038/s41419-025-07505-3)

Figure S1

Figure 3.

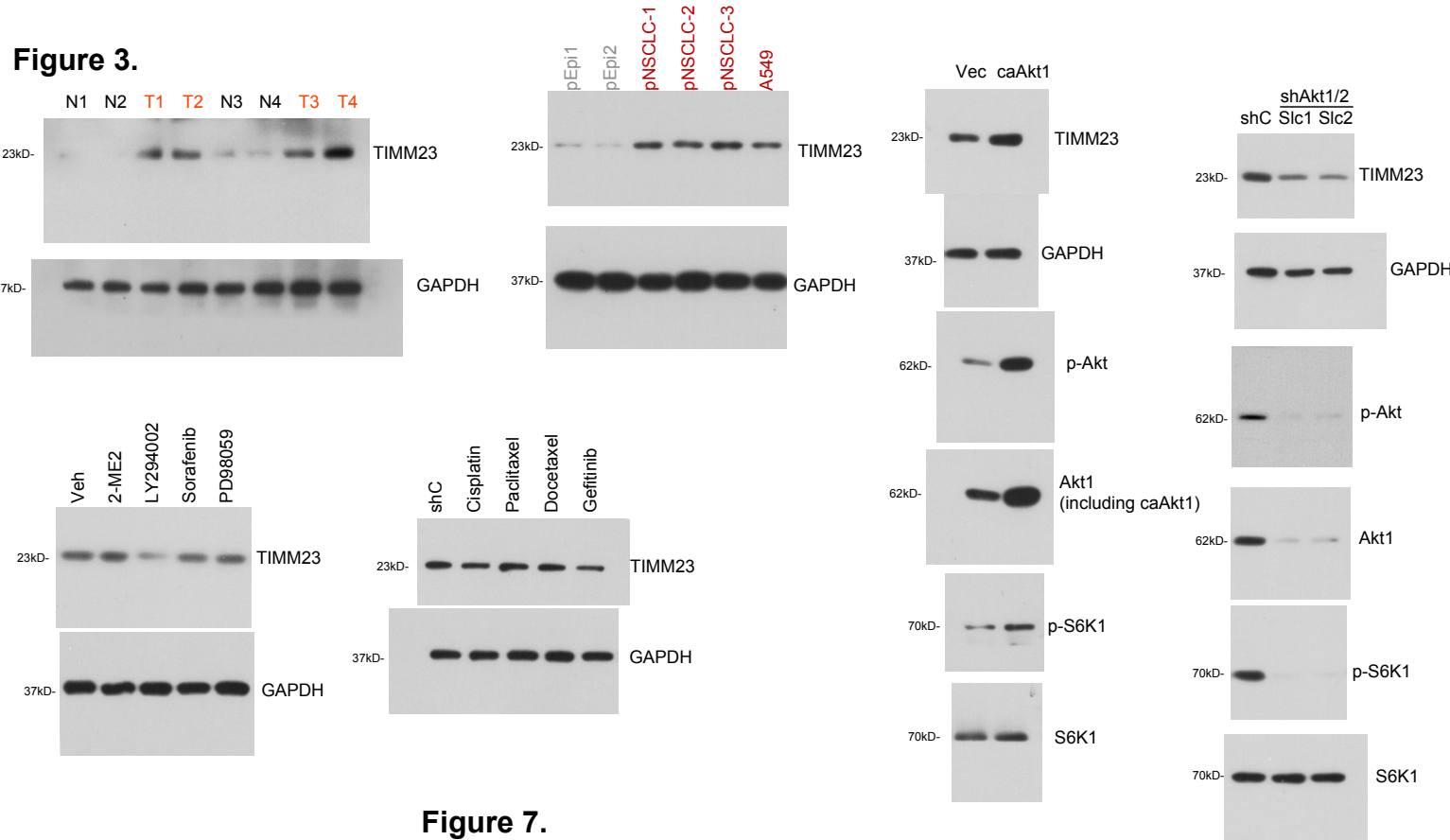

Figure 7.

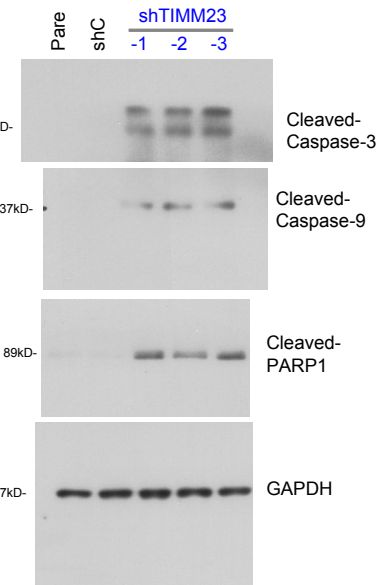

Figure 8.

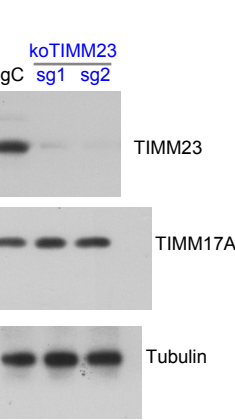

Figure 9.

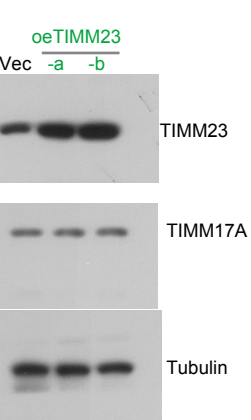

Figure 10.

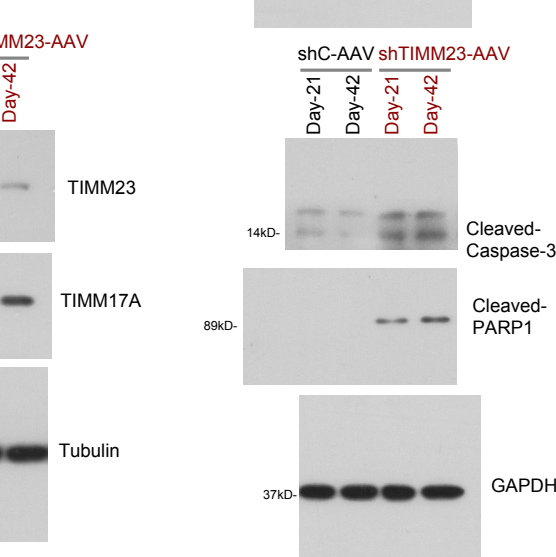

Figure S1. The un-cropped Western blotting images.

Supplement: Supplementary file 1 — Original data [file 41419_2025_7505_MOESM1_ESM.pdf]
